# Emerging Neuromodulation, Electrical Stimulation, and Cellular Therapies for Adult Cervical Spinal Cord Injuries in the United States: A Scoping Review

**DOI:** 10.7759/cureus.115549

**Published:** 2026-08-31

**Authors:** Marissa G Castronovo, Annalisa Peburn, Aliya Atcha, Joshua Singh, Naman Gupta, Srivennela Veeramachaneni, Sahil Soota, Samantha Scott, Audra Koechle, Patrick E Barry

**Affiliations:** 1 Osteopathic Medicine, Nova Southeastern University Dr. Kiran C. Patel College of Osteopathic Medicine, Davie, USA

**Keywords:** cellular therapies, cervical spinal cord injury, exercise-based rehabilitation, functional electrical stimulation, neuromodulation therapies, non-surgical treatments, pharamacologic treatment, scoping review

## Abstract

A variety of nonsurgical modalities are used in the management of cervical spinal cord injuries (CSCIs). However, comparative analyses across these treatment modalities remain limited, hindering the development of standardized care protocols. This scoping review aimed to map the existing literature on nonsurgical interventions for CSCIs and identify trends, fundamental principles, and key gaps to inform clinical practice and future research. A scoping review was conducted in accordance with Preferred Reporting Items for Systematic Reviews and Meta-Analyses Extension for Scoping Reviews (PRISMA-ScR) guidelines. EMBASE, Ovid Medline, Web of Science Core Collection, and Google Scholar were systematically searched using predefined criteria. Articles were screened using Rayyan. Studies published in English between January 2004 and September 2024 involving adult patients with CSCIs and conducted in the United States were included. Sixteen studies met the inclusion criteria. Data were extracted and synthesized based on predefined parameters, and a Preferred Reporting Items for Systematic Reviews and Meta-Analyses (PRISMA) flow diagram was generated. Identified interventions included neuromodulation, electrical stimulation, cellular therapies, pharmacologic treatments, and exercise-based rehabilitation. Most included studies reported improvements in neurophysiological, motor, or functional outcomes. However, findings varied across interventions and largely came from small studies with heterogeneous populations, protocols, and outcome measures. These findings map the current landscape of nonsurgical therapies for CSCIs rather than establish comparative treatment effectiveness. Although several interventions showed preliminary signals of benefit, the limited volume and heterogeneity of U.S.-based evidence preclude firm conclusions about treatment effectiveness.

## Introduction and background

Management of cervical spinal cord injuries (CSCIs) is complex and highly individualized, with treatment strategies and prognosis varying according to injury severity, level, and patient-specific factors. Variability in spontaneous recovery further complicates the establishment of a standardized treatment approach [1]. Approximately half of all spinal cord injuries (SCIs) occur at the cervical level, most commonly at C5 [2]. The lower cervical spine (subaxial cervical spine) is particularly prone to injury because of its greater mobility and transition to the more rigid thoracic spine [3]. CSCIs commonly result from motor vehicle collisions, falls, and sports-related injuries, particularly in younger populations. Secondary injury mechanisms, including inflammation, ischemia, edema, and neuronal hyperexcitability, further contribute to neurologic dysfunction [4]. 

CSCIs frequently impair upper extremity function, respiration, autonomic regulation, and mobility, resulting in substantial long-term disability and reduced quality of life. Recovery depends on both the initial mechanical injury and secondary injury processes, making treatment particularly complex [4]. Following SCIs, alterations in corticospinal and spinal network excitability may contribute to impaired motor function and represent potential targets for neuromodulatory rehabilitation [5]. 

Nonsurgical management is typically multimodal and may include pharmacologic therapy, rehabilitation, neuromodulation, assistive technologies, and other interventions aimed at preserving neurologic function, reducing complications, and maximizing functional recovery and quality of life [6]. In contrast, unstable injuries involving vertebral displacement are more likely to require surgical intervention [7]. Current treatment priorities include early stabilization, preservation of neurologic function, prevention of secondary injury, and initiation of rehabilitation to preserve quality of life [8].

However, variability in spontaneous recovery and the rapid evolution of nonsurgical therapies have limited the development of standardized, evidence-based treatment protocols [9]. Although growing research has expanded available treatment options, no clear consensus exists regarding optimal nonsurgical management, and many studies emphasize motor outcomes while comparatively few evaluate quality of life and long-term function. These gaps underscore the need for a comprehensive synthesis of the available evidence.

Nonsurgical interventions for CSCIs include both invasive and noninvasive approaches. For the purposes of this review, an invasive procedure will be defined as any procedure that involves deliberate access to the body via percutaneous puncture or incision [10]. Invasive modalities include stem cell therapies, functional electrical stimulation, and immunomodulation [11-13]. Noninvasive strategies include physical therapy, rehabilitation robotics and assistive devices, pharmacologic management, transcutaneous electrical nerve stimulation (TENS), and osteopathic manipulative treatment (OMT) [14-18]. 

Management of CSCIs is multidisciplinary. During the acute phase, immobilization is recommended to prevent further injury, followed by rehabilitation interventions that may improve mobility, strength, and neuropathic pain. Combined approaches, such as exercise and neuromodulation, may further enhance neuroplasticity, the nervous system's ability to reorganize surviving neural pathways and establish new functional connections following injury. Adjunctive considerations, including hemodynamic stability and preservation of autonomic function, remain critical throughout both acute and chronic phases of care [19-21].

Several commonly studied nonsurgical modalities, including chiropractic or manual therapy, acupuncture and other traditional medicine approaches, transcranial direct current stimulation (tDCS), peripheral stimulation, vibration therapy, constraint-induced movement therapy, virtual or augmented reality rehabilitation, robotic and exoskeletal technologies, hyperbaric oxygen therapy, aquatic therapy, and functional electrical stimulation (FES)-based exercise, were not represented among the eligible U.S.-based studies.

This scoping review aimed to map the current United States literature on nonsurgical interventions for adult CSCIs, with a focus on functional recovery, quality of life, and long-term outcomes.

## Review

Methods

Objectives

This scoping review aimed to systematically explore and map the existing literature on nonsurgical treatments for CSCIs for adults in the United States, with the goal of identifying trends, key concepts, and gaps in evidence. 

Rationale 

To our knowledge, no prior scoping review has comprehensively mapped nonsurgical treatment options specifically for adult CSCIs in the United States. This is largely due to the rapid emergence of novel therapies and the limited availability of long-term outcome data. A consolidated review of current treatment modalities may help inform clinical decision-making and support the development of more individualized, patient-centered strategies. 

*Eligibility Criteria* 

Studies were included if they were peer-reviewed, published in English within the past 20 years (between January 2004 and September 2024), conducted in the United States, and included adult participants with CSCIs. Eligible studies examined nonsurgical treatment modalities, including but not limited to orthobiologics, functional electrical stimulation, pharmacologic therapies, TENS, physical therapy, and OMT, across outpatient, inpatient, and specialized care settings.

Studies involving surgical interventions, defined as procedures requiring deliberate incision and general anesthesia, were excluded, as well as studies involving pediatric populations. Review articles (including systematic and scoping reviews) and animal studies were also excluded.

Information Sources 

EMBASE, Ovid MEDLINE, Web of Science Core Collection, and Google Scholar were searched to identify potentially relevant studies published between January 2004 and September 2024.

Search Strategy

A preliminary search of EMBASE (Table 1), Ovid MEDLINE (Table 2), and Web of Science Core Collection (Table 3) was conducted on September 13, 2024. Additionally, Google Scholar was searched using combinations of the primary search concepts to identify additional potentially relevant studies. Search strategies were developed using keywords and index terms related to cervical spinal cord injury, conservative treatment, adults, and the United States, combined using Boolean operators (“AND,” “OR”). 

**Table 1:** EMBASE – 09/14/2024. Results (number of articles identified): 8

Step	Search Query	Results
1	cervical spinal cord injury'/exp AND (2004-2024)/py	3897
2	('cervical spinal cord injury'/exp OR 'cervical spinal cord injur*':ab,ti,kw OR 'cervical cord injur*':ab,ti,kw OR 'cervical spinal cord lesion*':ab,ti,kw OR 'cervical spinal cord trauma':ab,ti,kw OR 'cervical spine cord lesion*':ab,ti,kw OR 'injur*, cervical spinal cord':ab,ti,kw OR 'injur*, spinal cord, cervical':ab,ti,kw OR 'spinal cord injur*, cervical':ab,ti,kw OR 'trauma*, cervical spinal cord':ab,ti,kw) AND (2004-2024)/py	4657
3	(#1 OR #2) AND (2004-2024)/py	4656
4	conservative treatment' AND (2004-2024)/py	111,951
5	('conservative treatmen*' OR 'nonsurgical invasive therap*' OR 'stem cell transplan*' OR 'stem cell based therap*' OR 'stem cell therap*' OR 'platelet rich plasma cell' OR 'prp' OR 'platelet-rich plasma cell' OR 'hyaluronic acid' OR 'regenerative medicine' OR 'bone marrow aspira*' OR 'bm aspirat*' OR 'functional electrical stimulation' OR 'transcutaneous electrical nerve stimulation' OR 'osteopathic manipulative procedur*' OR 'osteopathic manipulative technique*' OR 'osteopathic manipulative therap*' OR 'osteopathic manipulative treatmen*' OR 'osteopathic manipulatio*' OR 'physical therap*' OR 'physical treatmen*' OR 'physiotherap*' OR 'ferroptotic cell death' OR 'ferroptotic death' OR 'ferroptosis' OR 'robot-assisted rehabilitation device*' OR 'robotic rehabilitation device*' OR 'rehabilitation robot*' OR 'pharmacology' OR 'pharmacol*' OR 'pharmacological processes' OR 'pharmacognostics' OR 'immunomodulation' OR 'immune modulation' OR 'neuromodulation':ab,ti,kw) AND (2004- 2024)/py	3,330,090
6	(#4 OR #5) AND (2004-2024)/py	3,330,090
7	(#3 OR #6) AND (2004-2024)/py	832
8	adult*':ab,ti,kw AND (2004-2024)/py	1,825,882
9	('u.s.' OR 'u.s.a.' OR 'united states of america' OR usa OR 'united states':ab,ti,kw) AND (2004-2024)/py	3,980,235
10	(#8 OR #9) AND (2004-2024)/py	280,372
11	(#7 OR #10) AND (2004-2024)/py	8

**Table 2:** Ovid MEDLINE(R) ALL – 09/14/2024. Results (number of articles identified): 87

Step	Search Query	Results
1	exp Spinal Cord Injuries/	57797
2	limit 1 to yr="2004 -Current"	35494
3	('cervical spinal cord injur*' or 'cervical cord injur*' or 'cervical spinal cord lesion*' or 'cervical spinal cord trauma' or 'cervical spine cord lesion*' or 'injur*, cervical spinal cord' or 'injur*, spinal cord, cervical' or 'spinal cord injur*, cervical' or 'trauma*, cervical spinal cord').ab,ti,kf.	2853
4	limit 3 to yr="2004 -Current"	2132
5	2 or 4	36161
6	exp Conservative Treatment/	5451
7	limit 6 to yr="2004 - 2024"	5451
8	('conservative treatmen*' or 'nonsurgical invasive therap*' or 'stem cell transplan*' or 'stem cell based therap*' or 'stem cell therap*' or 'platelet rich plasma cell' or 'prp' or 'platelet-rich plasma cell' or 'hyaluronic acid' or 'regenerative medicine' or 'bone marrow aspira*' or 'bm aspirat*' or 'functional electrical stimulation' or 'transcutaneous electrical nerve stimulation' or 'osteopathic manipulative procedur*' or 'osteopathic manipulative technique*' or 'osteopathic manipulative therap*' or 'osteopathic manipulative treatmen*' or 'osteopathic manipulatio*' or 'physical therap*' or 'physical treatmen*' or 'physiotherap*' or 'ferroptotic cell death' or 'ferroptotic death' or 'ferroptosis' or 'robot-assisted rehabilitation device*' or 'robotic rehabilitation device*' or 'rehabilitation robot*' or 'pharmacology' or 'pharmacol*' or 'pharmacological processes' or 'pharmacognostics' or 'immunomodulation' or 'immune modulation').mp. or 'neuromodulation'.ab,ti,kf. [mp=title, book title, abstract, original title, name of substance word, subject heading word, floating sub-heading word, keyword heading word, organism supplementary concept word, protocol supplementary concept word, rare disease supplementary concept word, unique	4,169,020
	identifier, synonyms, population supplementary concept word, anatomy supplementary concept word]	
9	limit 8 to yr="2004 -Current"	2,178,226
10	7 or 9	2,178,226
11	("adult" and "united states").mp. [mp=title, book title, abstract, original title, name of substance word, subject heading word, floating sub-heading word, keyword heading word, organism supplementary concept word, protocol supplementary concept word, rare disease supplementary concept word, unique identifier, synonyms, population supplementary concept word, anatomy supplementary concept word]	221,995
12	limit 11 to yr="2004 -Current"	160958
13	"adult".mp. and ("united states" or "u.s." or "u.s.a." or "united states of america" or "usa" or "united states").ab,ti,kf. [mp=title, book title, abstract, original title, name of substance word, subject heading word, floating sub-heading word, keyword heading word, organism supplementary concept word, protocol supplementary concept word, rare disease supplementary concept word, unique identifier, synonyms, population supplementary concept word, anatomy supplementary concept word]	245499
14	limit 13 to yr="2004 -Current"	191085
15	12 or 14	251947
16	5 and 10 and 15	87

**Table 3:** Web of Science Core Collection – 09/15/2024. Results (number of articles identified): 1

Step	Search Query	Results
1	TS=(“cervical spinal cord injur*” or “cervical spinal cord lesion*” or “cervical spinal cord trauma*” or “injur*, cervical spinal cord” or “injur*, spinal cord, cervical” or “spinal cord injur*, cervical” or “trauma*, cervical spinal cord”) and PY = 2004-2024	2095
2	TS=("conservative treatmen*" or "nonsurgical invasive therap*" or "stem cell transplan*" or "stem cell based therap*" or "stem cell therap*" or "platelet rich plasma cell" or "prp" or "platelet-rich plasma cell" or "hyaluronic acid" or "regenerative medicine" or "bone marrow aspira*" or "bm aspirat*" or "functional electrical stimulation" or "transcutaneous electrical nerve stimulation" or "osteopathic manipulative procedur*" or "osteopathic manipulative technique*" or "osteopathic manipulative therap*" or "osteopathic manipulative treatmen*" or "osteopathic manipulatio*" or "physical therap*" or "physical treatmen*" or "physiotherap*" or "ferroptotic cell death" or "ferroptotic death" or "ferroptosis" or "robot-assisted rehabilitation device*" or "robotic rehabilitation device*" or "rehabilitation robot*" or "pharmacology" or "pharmacol*" or "pharmacological processes" or "pharmacognostics" or "immunomodulation" or "immune modulation” or “neuromodulation”)and PY = 2004-2024	705,261
3	#1 AND #2	167
4	TS=(“u.s” or “u.s.a” or “united states of america” or “usa” or “us” or “united states”) and PY = 2004-2024	1,970,664
5	TS=(“adult*”) and PY = 2004-2024	1,783,784
6	TS= #4 and #5	160,945
7	#3 and #6 and PY = 2004-2024	1

Study Selection 

All identified citations were managed using EndNote (Clarivate, London, United Kingdom), and duplicates were removed prior to screening. Titles and abstracts were independently screened by two reviewers, with discrepancies resolved by consensus. Full-text reports were subsequently assessed for eligibility by two independent reviewers, with a third reviewer serving as a mediator when necessary.

A total of 476 records were identified, of which 36 duplicates were removed, leaving 440 records for title and abstract screening. Following screening, 388 records were excluded, and 52 reports were sought for retrieval. One report could not be retrieved, leaving 51 reports for full-text assessment. Nineteen studies were selected for data extraction and critical appraisal. Following critical appraisal, three studies were excluded, resulting in 16 studies included in the final review. The study selection process is summarized in the PRISMA-ScR flow diagram (Figure 1).

**Figure 1:**
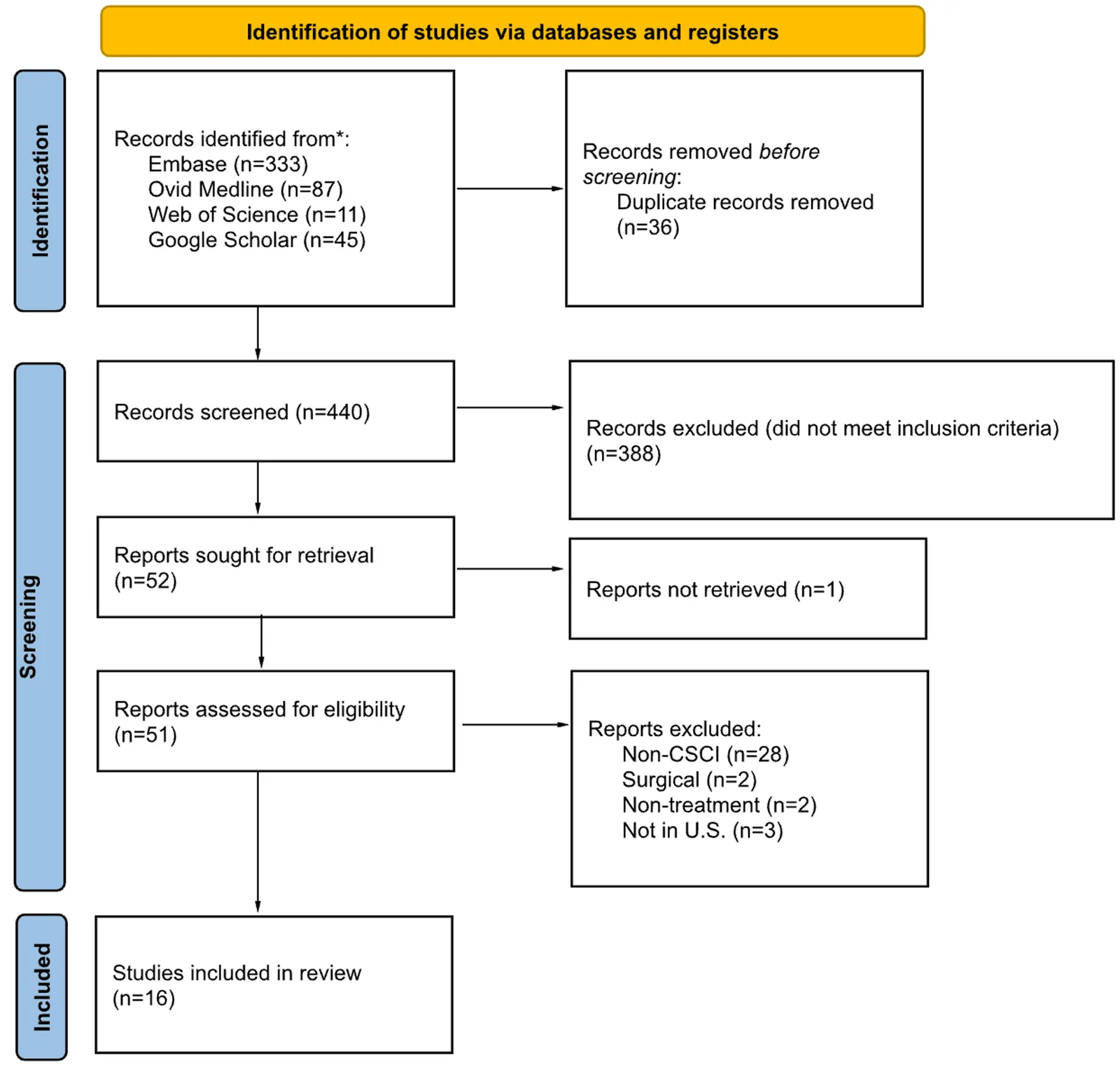
PRISMA diagram PRISMA flow diagram illustrating study identification, screening, eligibility assessment, and inclusion. A total of 476 records were identified across three databases, 36 duplicates were removed, 52 articles underwent full-text review, and 16 studies met the inclusion criteria. PRISMA: Preferred Reporting Items for Systematic Reviews and Meta-Analyses; CSCI: cervical spinal cord injury

Data Extraction 

Data were extracted and organized to capture the following parameters: study title, authors, country of origin, study aims, population and sample size (if applicable), methodology, intervention type and duration, comparator (if applicable), outcome measures, key findings relevant to the review question, study limitations, and treatment category.

*Data Analysis* 

Following data extraction, interventions were grouped into thematic categories based on similarities in therapeutic mechanism, treatment characteristics, and reported outcomes. Findings were synthesized descriptively across intervention categories. Two reviewers independently compared extracted study characteristics and grouped interventions according to therapeutic mechanism, treatment characteristics, and reported outcomes. Disagreements regarding thematic categorization were resolved through discussion and consensus.

*Quality Assessment (Critical Appraisal)* 

A critical appraisal was conducted using the Joanna Briggs (JBI) Critical Appraisal Tools [22] to assess methodological quality and risk of bias among studies selected for data extraction. The 19 studies that advanced to critical appraisal were independently assessed by two reviewers using the appropriate JBI checklists for randomized controlled trials and case series. For this review, studies were categorized as high risk of bias (<50%), moderate risk (50-70%), or low risk (>70%) based on appraisal scores.

Following independent assessment, discrepancies were reviewed by additional reviewers and resolved through discussion and consensus. Three studies were excluded following critical appraisal, resulting in 16 studies included in the final review. Of the 16 included studies, 15 were randomized controlled trials and one was a case series. All 16 included studies scored above the prespecified threshold for low risk of bias (>70%) (Table 4).

**Table 4:** Characteristics of included studies Characteristics of the 16 included United States-based studies evaluating nonsurgical interventions for adult CSCIs. The table summarizes study design, intervention type, sample size, comparator (where applicable), primary outcomes, key findings, and methodological limitations.

Title	Author(s)	Origin/country of origin	Aims/purpose	Study population and sample size (if applicable)	Methodology/methods	Intervention type/duration, comparator, outcome measures (if applicable)	Key findings that relate to scoping review questions	Limitations of the study	Treatment class
Acute intermittent hypoxia boosts spinal plasticity in humans with tetraplegia [23]	Lasse Christiansen, Bing Chen, Yuming Lei, M.A. Urbin, Michael S.A. Richardson, Martin Oudega, Milap Sandhu, W. Zev Rymer, Randy D. Trumbower, Gordon S. Mitchell, Monica A. Perez	United States	Examining whether paired corticospinal- motoneuronal stimulation (PCMS) could be potentiated by acute intermittent hypoxia (a treatment known to induce spinal cord injury (SCI) synaptic plasticity), the main question of this study was how to enhance the effect of PCMS to maximize recovery in humans with SCI	16 spinal cord injured participants (mean 50.2 +/- 12.8 y/o, 4 of whom were female). All participants had chronic SCI and were C1-C7 SCI based on ISNCSCI. Three individuals were AIS A, 8 were AIS C, and 5 were AIS D. Control subjects were age-matched and healthy.	PCMS: 180 pairs of stimuli in corticospinal volleys evoked by transcranial magnetic stimulation, PCMS was applied using a paired-pulse paradigm. EMG recorded from the first dorsal interosseous. Participants were seated with both arms relaxed and flexed at the elbow to 90 degrees with the forearm pronated and wrists/forearm restricted with straps. Participants had two sessions in random order separated by one week.	PCMS +AIH and PCMS+ sham-AIH (control) were the two random session orders. There was a baseline EMG recording before and after each session. outcomes measured with EMG (MEPs and MVC changes)	Acute intermittent hypoxia (AIH) is an effective strategy to enhance PCMS-induced spinal synapse plasticity. MEPs evoked by magnetic and electrical stimulation were increased when PCMS was combined with AIH therapy.	PCMS +AIH potentiated physiological outcomes but not voluntary motor control (needs to be considered when considering combo therapy).	Acute intermittent hypoxia
Efficacy and time course of acute intermittent hypoxia effects in the upper extremities of people with cervical spinal cord injury [24]	Milap S. Sandhu, Monica A. Perez, Martin Oudega, Fordon S. Mitchell, William z. Rymer.	Chicago (United States) and Northwestern University	The goal of this research was to investigate changes in hand strength and upper limb function elicited by episodic hypoxia and to establish how long these effects were sustained in persons with incomplete cervical SCI.	14 individuals with chronic, incomplete cervical SCI levels C4-C7	Randomized, blinded, placebo-controlled, and crossover design study consisting of a single AIH or SHAM AIH session. Outcome measures were recorded prior to and for up to 5 hours post AIH and on the next day. No sham AIH protocol is given in the article.	AIH protocol: single AIH sequence administered using a hypoxia generator with 60 seconds of 9% O2 with alternating 60 seconds of 21% O2. Hypoxia and normoxic air was provided alternating 15 x for a total of 30 minutes. Outcomes measured with hand dynamometry and clinical performance tests such as grip strength and pinch strength, nine-hole peg test, and box and block test	AIH therapy is a promising technique in eliciting spinal plasticity and drive functional recovery. A single AIH session enhances b/l grip and pinch strength, an effect that peaks 3 hours post AIH. Main finding: AIH enhances hand strength and function in persons with cervical SCI by potentiating corticospinal excitability.	Single AIH sequence, sample size small, only followed patients 1 day following administration of AIH sequence, paper does not include the protocol for the placebo group.	Acute intermittent hypoxia
Effects of acute intermittent hypoxia on hand use after spinal cord trauma: A preliminary study [25]	Randy D. Trumbower, Heather B. Hayes, Gordon S. Mitchell, Steven L. Wolf, Victoria A. Stahl	United States	Test is daily acute intermittent hypoxia (AIH) combined with hand opening practice improves hand dexterity, function, and maximum hand opening in persons with cervical SCI	Six patients with chronic, motor incomplete cervical SCI	Patients received 1.5 minutes of AIH 15 times a day followed by 20 reps of hand opening practice with normoxia for 5 days. Maximum hand opening measured by motion analyses and co-activity of extensor digitorum and flexor digitorum superficialis using surface EMG	Double blind crossover study. Hand dexterity and function were quantified with Box and Block (BBT) and Jebsen-Taylor (JTHF) hand function tests	Daily AIH plus hand practices improved hand dexterity, function, and maximum hand opening in all participants. BBT scores improved in 5 participants compared to baseline. All participants reduced their JTHF times. In 5 participants, there was improved EMG coactivity	Small sample, no analysis of differences in use responsiveness based on medication use, severity and time since injury	Acute intermittent hypoxia
Improvements in hand function in adults with chronic tetraplegia following a multiday 10-Hz repetitive transcranial magnetic stimulation intervention combined with repetitive task practice [26]	Joyce Gomes-Osman, Edelle C. Field-Fote	United States	Assessed effects of a multiday application of 10-Hz repetitive transcranial magnetic stimulation (rTMS) applied to the corticomotor hand area combined with repetitive task practice (RTP) in participants with tetraplegia and neurologically healthy participants	13 participants with chronic tetraplegia at level C7 or more and 10 neurologically healthy participants	3 sessions of 10 Hz rTMS +RTP and 3 sessions of sham rTMS+RTP to the corticomotor hand region controlling the weaker hand over 3 days	Double blind, randomized, crossover design. Jebsen-Taylor hand function test (JTT) and corticomotor excitability were assessed before and after rTMS+RTP phases	rTMS+RTP had a larger effect (almost twice as large) on the improvement in JTFT for both the trained and non-trained hand, and for grip strength of the trained hand. Median changes in JTT time met the minimal clinically important differences (MCID), meaning most participants made improvements in hand function when they engaged in rTMS+RTP.	2 participants discontinued participation before the end of the study- one family emergency, one no reason. Statistically significant differences between the normal and the sham group were not found. Possible ceiling effect for performance of routine motor task	Electrical stimulation
Intermittent theta burst stimulation modulates biceps brachii corticomotor excitability in individuals with tetraplegia [27]	Neil Mittal, Blaize C. Majdic and Carrie L. Peterson	United States	Determine the effect of intermittent theta burst stimulation (ITBS) on the corticomotor excitability of the biceps brachii in individuals with tetraplegia	Ten individuals with low cervical (C5-C8) SCI and ten nonimpaired individuals	Individuals in the study completed three independent sessions. Motor evoked potentials were the measure of corticomotor excitability of the BB before and after ITBS. Each session was separated by 3 days to prevent potential carryover from one session to another. Patients were seated in a chair with their dominant arm at rest.	Each participant completed 3 independent sessions of the iTBS protocol, yielding 30 independent sessions in the nonimpaired group and 30 independent sessions in the SCI group. EMG was recorded from the long head of the biceps and lateral head of the triceps. iTBS comprised three pulses at 50 Hz repeated every 200 ms for 2 s at an intensity of 80 % of the participant's AMT. Two-second bursts were repeated every 8 seconds for a total of 600 pulses.	There was an increase in nMEP (normalised MEP); results indicated that the biceps brachii is responsive to iTBS for increased corticomotor excitability in individuals with tetraplegia. Study findings conclude that ITBS would be a viable adjunct therapy with physical therapy for motor rehabilitation	Could not assess biceps corticomotor excitability at stimulus intensities of 120% RMT, resulting in potential understimulation. Article gives a suggestion for addressing this limitation by utilizing a monophasic stimulator to evaluate RMTs and collect MEPs.	Electrical stimulation
Corticospinal-motor neuronal plasticity promotes exercise-mediated recovery in humans with spinal cord injury [28]	Hang Jin Jo, Monica A. Perez	United States	Determine if exercise combined with non-invasive stimulation targeting spinal synapses promotes functional recovery	38 with chronic, incomplete cervical, thoracic, or lumbar SCI	Randomized complete block design. 25 patients complete 10 sessions of paired corticospinal-motor neuronal stimulation (PCMS) or sham-PCMS followed by 45 minutes of exercise. 13 patients do PCMS without exercise. 6-month follow-up session	Measures motor evoked potential (MEP) and maximal voluntary contraction (MVC) before and after each intervention. EMGs recorded from the deltoid, biceps brachii, 1st dorsal interosseous, abductor pollicis brevis, and tibialis anterior. In PCMS, 180 pairs of stimuli were delivered every 10 seconds. Outcomes measured: gross and fine grasping functions, 10m walk time.	Amplitude of corticospinal responses and magnitude of maximal voluntary contractions increased by 40-50% after PCMS and exercise, or without exercise. No differences were observed in the sham-PCMS group	Sample size, no long-term follow-up, didn't use different stimulation intensities	Physical therapy
Cortical and Subcortical Effects of Transcutaneous Spinal Cord Stimulation in Humans with Tetraplegia [29]	Francisco D. Benavides, Hang Jin Jo, Henrik Lundell, V. Reffie Edgerton, Yuri Gerasimenko, and Monica A. Perez	United States	Examined motor-evoked potentials in arm muscles elicited by cortical and subcortical stimulation of corticospinal axons before and after 20 minutes of Transcutaneous electrical stimulation of the spinal cord TESS	17 individuals with C4-C6 SCI and 15 age-matched control subjects. 4 AIS A, 3 AIS B, 2 AIS C, 8 AIS D	Single blind. 20 minutes of transcutaneous electrical stimulation of the spinal cord (TESS) or sham-TESS applied between C5 and C6 spinous processes. MRI used to ensure TESS was applied over injured segments. Monitored effect on motor evoked potentials (MEP) and cervicomedullary electrical stimulation (CMEP)	EMG recorded from the biceps brachii muscle of the right side in control subjects and from the less affected arm. TESS sessions separated by 2-3 days. Stimulation was delivered between C5-C6. Tests were done with and without a 5 kHz carrier frequency. Outcomes measured by MEP and CMEP SICI and functional outcomes measured before, immediately after and 75 minutes after.	TESS can change the excitability of cortical and spinal neuronal networks in uninjured and SCI humans. size of CMEPs not MEPS increased in proximal and distal arms. TESS was greatest when used with 5kHz frequency	Clearer understanding of the neuronal connectivity underlying the TESS-induced recovery may help maximize rehabilitation strategies.	Electrical stimulation
Improving upper extremity strength, function, and trunk stability using wide-pulse functional electrical stimulation in combination with functional task-specific practice [30]	Candace Tefertiller, Patricia Bartelt, Maureen Stobelaar, Susie Charlifue, Mitch Sevigny, Eric Vande Griend, and Megan Rozwod	United States	Evaluate UE function, strength, and dynamic sitting balance in individuals with SCI who received an intensive outpatient therapy program focused on UE training augmented with wide pulse/high frequency functional electrical stimulation	50 patients with tetraplegia recieving OP therapy focused on UE recovery Criteria: presence of cervical spinal cord lesion, previous completion of inpatient rehab, no use of botox a ability to achieve motor response to FES in the majority of UE muscle groups impacted by SCI, medical referral by a physician for occupational therapy, ability to use electrically evoked contraction of the should to maintain humeral head in GH joint, ISNCSCI completed within 30 days of study enrollment.	Prospective case series conducted in an outpatient clinic in an SCI-specific rehab hospital	Outcome was the capabilities of the upper extremity test (CUET), assesses the amount of difficulty experienced in performing specific actions with one or both arms and hands. pts participated in 60 minutes of UE functional task-specific practice (FTP) in combination with WPHF-FES 5 x a week for an average of 72 sessions. The primary outcome was CUET; the secondary outcome was UEMS and modified functional reach (MFR). UE training had three components: 60 min of FTP in combination with WPHF-FES to the trunk and UEs 15 min of functional training without WPHF-FES and home integration training.	The primary outcome was CUET; the secondary outcome was UEMS and modified functional reach (MFR). Participants demonstrated significant improvements in the total CUET score and improvements were noted in the UEMS and MFR scores. CUET score improved by an average of 14.1, UE motor score improved by an average of 4.6, and MFR improved by an average of 13.6. Significant positive association with the number of UE training sessions completed and CUE-T total score; each additional training session increased the change by 0.15 CUET points.	Lack of control group, low sample size in complete SCI group, missing data for secondary outcome measures	Electrical stimulation
EEG-controlled functional electrical stimulation for hand opening and closing in chronic complete cervical spinal cord injury [31]	Katie Gant, Santiago Guerra, Lauren Zimmerman, Brandon A. Parks, Noeline W. Prins, Abhishek Prasad	United States	Evaluate brain-computer interface -functional electrical stimulation system (BCI-FES system) in SCI subjects with chronic cervical injuries and compare them to uninjured subjects. This study was started because patients with tetraplegia ranked restoration of their hand and arm function as a primary goal in maintaining quality of life.	8 patients with C5 or C6 chronic motor complete SCI. Control: 6 healthy uninjured patients	6 sessions of brain computer interface-functional electrical stimulation (BCI-FES) over 2-7 weeks. Subjects would "imagine moving hand"; the computer followed eye movements and EEG signals, and a current was sent to flexor or extensor muscles in the hand. EEG was recorded continuously during sessions, also noted that the muscle stimulation program was custom and was developed for the purpose of opening and closing hands (grasp release motions). Muscles targeted were extensor digitorum profundus, extensor digitorum superficialis, extensor pollicis longus, flexor digitorum superficialis, and flexor digitorum profundus.	Intervention is BCI-FES for 6 sessions. Two groups: cervical SCI group with varying mechanisms of injury (listed in the paper) and control uninjured healthy individuals. outcomes measured by EEG	Average online decoding accuracy during the BCI-FES was similar for the SCI (74.8% +/- 17.76) and the control (75.5+/-11.94). Patients with SCI were able to modulate their EEG activity with similar accuracies compared to the controls. Also noted that SCI patients were more motivated and active in trying to master the BCI-FES training. SCI patients were able to control a BCI-FES with similar performance levels as control subjects.	Some subjects were better able to modulate their neural activity compared to others. Future studies should look at varying the channels fed to the decoder and optimizing features to obtain higher accuracies. Other considerations: functional benefits, cosmetic look, reliability, invasiveness, cost, burden of maintenance, requirement of caregiver or tech to help.	Electrical stimulation
A phase 1/2a dose-escalation study of oligodendrocyte progenitor cells in individuals with subacute cervical spinal cord injury [32]	Richard G. Fessler, MD, PhD, Reza Ehsanian, MD, PhD, Charles Y. Liu, PhD, MD, Gary K. Steinburg, MD, PhD, Linda Jones PT PhD, Jane S. Lebkowski PhD, Edward D. Wirth III Md PhD, Stephen L Mckenna MD	United States	Evaluate the safety of 3 escalating doses of oligodendrocyte progenitor cells (lineage cell therapeutics oligodendrocyte progenitor cells (LCTOPC1)) and changes in neurological function	C5-C7, 25 participants, Grade A, B AIS	1 or 2 injections administered 13-30 days post injury, open labeled staggered dose escalation multicenter phase 1/2a clinical trial. Patients were assigned to 1 of 5 cohorts with varying dosages and injections.	Grade A SCI received 1 of 3 sequential doses grade B SCI received 1 X 10 ^7 or 2x 10 ^7 LCTOPC1. All patients who received LCTOPC1 also received low-dose tacrolimus (immunosuppression. Participants were followed for 1 year. Measurements used to assess safety PE, vitals, ECG, neuroexams (ISNCSCI), MRIs, pain questionnaires, AEs and labs. Neurological function was assessed with ISNCSCI on all participants at 30, 60, 90, 180, 270, 265 days post final injection.	All 25 participants experienced at least 1 AE with the most common being urinary tract infection. Of the 534 AEs reported, 1 was related to the LCTOPC1 (grade 2 dysesthesia 47 post injection and resolved by two-year follow-up), 20 related to the injection procedure, and 11 related to tacrolimus. Neurological function changes following LCTOPC1: baseline UEM ISNCSCI score was 28.4 in 22 participants, with a mean improvement from baseline by 8.9 in one year following completion of injection. Cohort 2 had the best improvement in ISNCSCI score with mean 12.3 score increase. Cohort 2 treatment plan had 1 LCTOPC1 injection of 1 x10^7 #cells with [2x10^5].	Sample size is a limitation noted from this study suggestion of subfascial drainage with collection bags due to patients with post-operative spinal cord compression due to CSF buildup. This study was not powered to evaluate neurological improvement regarding LCTOPC1 injection.	Biologics
A prospective, multicenter, Phase i matched-comparison group trial of safety, pharmacokinetics, and preliminary efficacy in riluzole in patients with traumatic spinal cord injury [33]	Robert G. Grossman, Michael G. Fehlings, Ralph F Frankowski, Keith D. Burau, Diana S.L. Chow, Charles Tator, Angela Teng, Elizabeth G. Toups, James S. Harrop, Bizhan Aarabi, Christopher I. Shaffrey, Michele M. Johnson, Susan J. Harkema, Maxwell Boakye, James D. Guest, Jefferson R. Wilson	United States	Investigate the pharmacokinetics and safety as well as obtain pilot data on the effects of riluzole on neurological outcome in acute spinal cord injury	36 patients grades A-C (28 cervical, 8 thoracic) C4-T11	Patients received 50 mg of riluzole PO/NG twice daily within 12 hours of injury for 14 days.	Multisite, single-arm, open-label treatment pilot study. Rates of medical complications, adverse effects, and progression of neurological status evaluated by comparison with matched patients in the NACTN SCI registry. Outcomes measured: prehospital demographic data, medical condition evaluation, details of medical and surgical therapy received, hematology and blood chemistries on days 3, 7, 10, 14, 42, 90, 180, medical complications and serious adverse events, and measurement of neurological status on days 3, 14, 42, 90, 180	Riluzole is a sodium channel blocker, approved by the FDA for ALS and has been shown to improve the outcome of SCI in preclinical studies. Peak plasma concentration and systemic exposure to riluzole varied significantly between patients. Mild to moderate increases in liver enzymes and bilirubin levels were found in 14-70% of patients. Three patients had borderline severe elevation of enzymes. There were no serious adverse effects or deaths. mean motor score of 24 in riluzole-treated patients gained 31.2 points from admission to 90 days, compared to 15.7 points for 26 registry patients. At 180 days, there was no significant difference in the spinal cord independence measure between the riluzole and registry groups	One patient had to withdraw because his liver function tests showed a moderate elevation of GGT. open label - possible positive bias. Factors other than treatment with riluzole could have influenced neurological outcome. Small sample size.	Pharmacology
Soluble Nogo-receptor-Fc Decoy in patients with chronic cervical spinal cord injury in the USA: a first-in-human and randomised clinical trial [34]	George Maynard, Ramakrishnan Kannan, Jian Liu, Weiwei Wang, Tu Kiet T Lam, Xingxing Wang, Crista Adamson, Craig Hackett, Jan M Schwab, Charles Liu, Donald P Leslie, David Chen, Ralph Marino, Ross Zafonte, Adame Flonders, Glibert Block, Erika Smith, Stephen M Strittmatter	United States	Previous animal studies found that Soluble Nogo-Receptor-Fc decoy(AXER-204) blocked inhibitors of Axon growth and promoted recovery of neural disconnection after SCI. This is the first-in-human, randomized clinical trial of the application of AXER-204 in individuals with chronic SCI and its effects on recovery.	24 patients in part 1 (six/dose 18 men 6 women), 27 patients in part 2 (13 placebo, 14 AXER-204)	Two-part study with chronic (>1 year) cervical SCI at six rehab centers in the USA. The first study was open-label with primary outcomes of safety and pharmacokinetics; the second study was a randomized, parallel, double blind comparison of 6 intrathecal injections of 200 mg AXER-204 vs placebo.	Part 1: single intrathecal doses of 3 mg, 30mg, 90 mg, or 200 mg of AXER-204 for safety and pharmacokinetic analysis. Part 2: six intrathecal doses of 200 mg AXER-204 or placebo over 104 days. Upper extremity motor score was analyzed on day 169 for all enrolled patients. Outcomes: headache was a common side effect; AXER-204 reached maximal CSF concentration 1 day after dosing with 200 mg. In part 2, the mean changes from baseline to day 169 in the International Standards for Neurological Classification of SCI upper extremity motor score were 1-5 for AXER-204 and 0-9(2-3) for placebo (mean difference 0-54, 95% CI -1*48 to 2*55; p=0.59)	There was no statistically significant difference between the AXER-204 and placebo groups in the change in ISCNCSCI UEMS or other efficacy outcome measures across all patients. However, subgroup analyses of patients with ASI grade B c or D who were not previously treated with AXER-204 in part 1 improved by 4 points in the UEM score and 9 points in the total score. Findings suggest that incomplete SCI is more amenable to therapy than complete SCI.	There was no statistically significant difference between the AXER-204 and placebo groups in the change of ISCNCSCI UEMS or other efficacy outcome measures across all patients *****	Biologics
Acute Ventilatory Support During Whole Body Hybrid Rowing in Patients with High Level Spinal Cord Injury [35]	Isabelle Vivodtzev, Glen Picard, Felipe Cepeda, Andrew Taylor	United States	Tested effects of noninvasive ventilation (NIV) on ventilatory and aerobic capacities in SCI.	19 patients range of injury C4-T8	Blinded, randomized crossover study. Patients were those who were enrolled in an outpatient rehab program that used hybrid FES-rowing training.	First session for familiarization, followed by 2 FES-rowing peak exercise tests with NIV or sham in random order. Patients received NIV or sham via BIPAP. A double-limb circuit used in both conditions with ventilatory on spontaneous mode. The primary outcome was the changes in peak alveolar ventilation and peak oxygen consumption during an incremental FES-rowing test. The secondary outcome was changes in respiratory pattern	Acute NIV can successfully improve ventilatory efficiency during FES exercise in SCI but may not improve VO2 peak in all patients. NIV significantly changed respiratory pattern. Training adaptations to whole-body FES rowing may be limited by reduced ventilatory capacity in individuals with high-level SCI; this study indicates that NIV therapy can alleviate the limitation of FES rowing in upper cervical spinal cord injury patients	Sample size, test effects of NIV in earlier staging of FES-rowing training rehab.	Ventilation
Increase in Muscle activation during physiotherapy with electromyography biofeedback for patients with acute cervical spinal cord injuries [36]	Guijin Li, Caitlin Doman, and Nina L Suresh	United States	Assess whether surface electromyography biofeedback display during PT sessions for acute SCI would assist with increasing muscle use and patient engagement during therapy	2 patients from Shirley Ryan Ability Lab with c3/c4 and c4/c5 injury	Control: participants repeat elbow flexion 10 times under conventional sling therapy. Biofeedback group: the therapist guided the patient in the same movement, but they were provided with visual feedback of real-time SEMG signals recorded from the biceps brachii muscles	6 sessions over several weeks with control performed on both patients, followed by biofeedback. The two patients were unable to complete the 6 sessions, for a variety of clinical reasons.	Average rectified values are higher in biofeedback than control	**Small sample size (2 patients); both patients were unable to complete the study also noted that biofeedback was not standardized for this experiment and bias was not prevented.	Electrical stimulation
Massed practice versus massed practice with stimulation: Effects on upper extremity function and cortical plasticity with incomplete cervical spinal cord injury [37]	Kristina S Beekhuizen, Edelle C. Field-Fote	United States	Determine the effect of massed practice (MP) versus mass practice combined with somatosensory stimulation (MP+SS) on cortical plasticity and function in persons with incomplete tetraplegia	10 participants, incomplete SCI of at least 1 year, injury at C7 or higher	Participants were assigned to either MP or MP+SS. Median nerve stimulation was delivered at the intensity to elicit a motor threshold response. Training sessions were 2 hours for 5 days a week for 3 weeks.	Outcomes measured: motor-evoked potentials (MEP) elicited via transcranial magnetic stimulation, motor threshold (MT), MEP amplitude, maximal pinch grip force, Wolf Motor Function Test (WMFT), Jebsen Hand Function Test	MS+SS group had statistically significant improvements in pinch grip, WMFT, and Jebsen test scores. No significant changes were detected in cortical excitability in the MP+SS or MP group	Only 5 subjects per group, only median nerve, further studies should include F wave studies to measure spinal level excitability.	Electrical stimulation
Non-invasive activation of cervical spinal networks after severe paralysis [38]	Parag Gad, Sujin Lee, Nicholas Terrafranca, Hui Zhong, Amanda Turner, Yury Gerasimenko, V. Reggie Edgerton	United States	Determine if transcutaneous enabling motor control (tEmc) is effective in neuromodulating the cervical spinal networks to improve upper limb motor function	8 patients with SCI AIS grade B and C	2 sessions a week of transcutaneous stimulation along with upper extremity strength training over 4 weeks. hand grip tasks were completed without stimulation, then with, and then without again for every session. Two stimulation sites were placed on each patient at the space between the spinous processes of C3-C4 and C6-C7	Baseline measurements: neurophysiological tests, hand grip strength. EMG recorded muscle activity from biceps brachia and flexor and extensor digitorium	Mean increase in grip strength ~325%, all subjects reported improvements in one or more measures of quality of life. At the end of the study grip strength with and without stimulation was increased. All patients reported improvements in finger and hand dexterity	Small sample size, there was no control group or blinded group. They did not use different frequencies or change the duration of stimulation	Electrical stimulation

Results

*Thematic Categories* 

The 16 studies included in the final review were categorized into four major thematic domains: (1) neuromodulation and neuroplasticity (2) electrical stimulation and assistive technologies, (3) biologic and pharmacologic therapies, and (4) rehabilitation and exercise-based interventions.

Neuromodulation strategies, including paired corticospinal-motoneuronal stimulation (PCMS), acute intermittent hypoxia (AIH), repetitive transcranial magnetic stimulation (rTMS), and intermittent theta burst stimulation (iTBS), primarily targeted corticospinal excitability and neuroplasticity. Studies evaluating AIH reported short-term improvements in upper extremity strength following single-session protocols, while studies combining AIH with task-specific training also reported improvements in functional outcomes [23-25]. Additional neuromodulatory approaches, such as rTMS and iTBS, further supported increases in corticomotor excitability and motor recovery [26,27]. Combination strategies, particularly PCMS paired with exercise, were associated with improvements in neurophysiological and functional outcomes [28].

Electrical stimulation modalities, including transcutaneous electrical spinal stimulation (TESS), FES, and transcutaneous enabling motor control (tEmc), were commonly used to improve motor activation and functional independence. TESS revealed both cortical and subcortical effects, while FES-based interventions were frequently incorporated into rehabilitation protocols [29,30]. Advanced technologies, such as BCI-FES, enabled restoration of coordinated motor tasks even in patients with complete CSCIs [31].

Biologic and pharmacologic interventions focused on neuroprotection and neural regeneration. Oligodendrocyte progenitor cell therapies were investigated for their potential to promote remyelination and neurological recovery, while pharmacologic agents such as riluzole and AXER-204 were investigated for their potential neuroprotective and functional effects [32-34].

Rehabilitation-based interventions, including task-specific training, massed practice, somatosensory stimulation, and surface electromyography (sEMG) biofeedback, were associated with improvements in strength, coordination, and functional performance. Hybrid approaches, such as FES-assisted exercise and non-invasive ventilation (NIV)-supported training, also addressed cardiovascular and respiratory limitations in this population [30,35-37].

Key Findings 

Across the included studies, many interventions were associated with improvements in physiological and functional outcomes, although the magnitude and consistency of these findings varied across interventions and study designs. Neuromodulation techniques increased motor-evoked potentials and corticospinal excitability, though translation to functional gains varied depending on patient characteristics and intervention protocols [23,27].

Studies evaluating AIH reported both acute and cumulative improvements. Single-session protocols optimized grip and pinch strength with peak effects several hours post-intervention, while repeated exposure and combination with task-specific training resulted in sustained improvements in hand function and dexterity [24,25].

Several studies evaluating combination therapies reported improvements in functional outcomes compared with single-modality or sham interventions; however, differences in study populations, intervention protocols, and outcome measures limited direct comparison across studies. PCMS combined with exercise was associated with greater and longer-lasting improvements in motor function than either intervention alone in the included study [28]. Similarly, stimulation-based approaches paired with rehabilitation, such as rTMS combined with repetitive task practice, were associated with greater improvements in strength and functional performance compared with sham intervention in the included study [26].

Electrical stimulation techniques, including FES and TESS, were associated with improvements in motor activation and functional capacity. FES combined with task-specific training enhanced upper extremity strength, trunk stability, and functional outcomes, while TESS enhanced both spinal excitability and upper limb function [29,30]. Additionally, tEMC was associated with improved hand grip strength and sensorimotor integration [38]. BCI-FES systems enabled patients with chronic, complete CSCI to perform coordinated reaching and grasping tasks [31].

The included biologic studies reported preliminary safety and functional outcome findings, although sample sizes were small and follow-up was limited. Oligodendrocyte progenitor cell transplantation resulted in motor recovery in a majority of participants, while riluzole was associated with higher motor scores without significant adverse effects [32,33]. AXER-204 was well tolerated, with greater functional improvement observed among patients with incomplete injuries [34].

Rehabilitation interventions, including massed practice and somatosensory stimulation, were associated with improvements in grip strength and upper extremity function, while sEMG biofeedback enhanced muscle activation compared to standard therapy alone [36,37].

*Identified Gaps* 

Several key limitations were identified across the literature. Most studies were limited by small sample sizes, reducing generalizability and limiting subgroup analyses based on injury severity, level, and chronicity. Despite the predominance of randomized controlled trials, methodological limitations, including incomplete blinding, variable control conditions, and differences in study design, were present across the literature.

There was considerable heterogeneity in intervention protocols, treatment duration, and outcome measures, making comparisons across studies difficult. Inconsistent definitions of functional improvement further limit the ability to establish standardized treatment efficacy.

Long-term follow-up data were largely absent, restricting assessment of durability and long-term clinical impact. Additionally, the mechanisms underlying several neuromodulatory therapies, particularly PCMS, AIH, and TESS, remain incompletely understood, limiting optimization of these interventions [23,29].

Variability in treatment response was influenced by patient-specific factors, including injury level, completeness, and time since injury. For example, the effectiveness of NIV-assisted exercise and pharmacologic therapies varied across patient populations [34,35].

Although AIH revealed promising neuromodulatory effects, its impact on upper extremity function appears more modest compared to lower extremity outcomes, and optimal dosing strategies and long-term efficacy remain unclear [24].

*Emerging Trends* 

The included literature reflects an increasing emphasis on multimodal and technology-assisted treatment strategies. Combination therapies integrating neuromodulation, electrical stimulation, and rehabilitation approaches were represented among the included studies and may warrant further investigation for potential additive effects [25,28].

Technological advancements represent a major area of innovation. BCI-FES systems show preliminary potential for restoring motor control and independence in patients with severe CSCI [31]. Additionally, the integration of FES with task-specific rehabilitation is emerging as a potentially beneficial approach to improve strength, coordination, and functional outcomes [30].

Overall, the included literature reflects increasing interest in individualized, multimodal approaches that combine biologic, technological, and rehabilitation-based interventions to potentially enhance recovery following CSCI.

Discussion 

Principal Findings

This scoping review mapped nonsurgical treatment modalities for adult CSCIs in the United States. Several interventions were associated with improvements in neurophysiological and functional outcomes. Although all included studies were classified as low risk of bias using the JBI Critical Appraisal Tools, the small sample sizes, heterogeneous methodologies, and varied outcome measures limit the strength and generalizability of the evidence. Accordingly, the findings identify areas of potential benefit but do not establish comparative effectiveness or support treatment recommendations.

A recurring feature of the included literature was the use of multimodal treatment strategies. PCMS combined with exercise and AIH paired with task-specific training were associated with improvements in functional outcomes, although differences in study populations and protocols limited direct comparison across interventions. AIH alone was associated with transient improvements in upper extremity strength. Electrical stimulation modalities, including FES, TESS, and tEmc, were associated with improvements in motor activation and functional performance.

Emerging technologies, particularly brain-computer interface (BCI)-integrated FES systems, showed preliminary potential for improving motor function in patients with severe or complete CSCIs. Biologic and pharmacologic therapies, including oligodendrocyte progenitor cell transplantation, riluzole, and AXER-204, also demonstrated preliminary safety and functional signals, although long-term effectiveness remains uncertain.

Comparison With Existing Literature

The included studies generally reflected findings reported in prior research on neuromodulation and electrical stimulation, although differences in study populations and protocols limit direct comparison. The included studies also reflected growing interest in combination therapies; however, differences in study design, patient populations, intervention protocols, and outcome measures limit conclusions regarding whether these approaches provide greater or more sustained benefits.

Evidence regarding the optimal timing, dosing, and sequencing of these interventions remains limited. Although several studies reported short-term benefits, particularly with neuromodulatory approaches such as AIH and rTMS, insufficient evidence exists to determine how these therapies should be integrated longitudinally into clinical care.

Gaps in the Literature and Future Directions

A major gap across the included studies was the lack of long-term follow-up, limiting assessment of sustained functional recovery and clinical impact. In addition, the underlying mechanisms and optimal parameters of several neuromodulatory therapies, including PCMS, AIH, and TESS, remain incompletely understood.

Future studies should prioritize multicenter randomized controlled trials with standardized intervention protocols, clinically meaningful functional and patient-reported outcome measures, and longer follow-up. Comparative studies of multimodal treatment strategies may help clarify how emerging therapies can be integrated into comprehensive CSCI rehabilitation. Broader evaluation of commonly used conservative therapies and international evidence would further strengthen the evidence base.

Strengths and Limitations of the Review

Although this review followed established JBI and PRISMA-ScR methodology, several limitations should be acknowledged. Inclusion was restricted to English-language studies conducted in the United States, limiting generalizability and excluding international evidence. Consequently, several commonly studied conservative therapies, including acupuncture, tDCS, virtual reality-assisted rehabilitation, and chiropractic care, were not represented in the included US-based literature. Although EMBASE, Ovid MEDLINE, Web of Science Core Collection, and Google Scholar were searched, additional studies indexed elsewhere may have been missed.

Consequently, the thematic emphasis on neuromodulation, electrical stimulation, and biologic therapies reflects the available United States evidence identified by our search rather than the relative importance or frequency of these interventions in routine clinical practice.

Many included studies lacked long-term follow-up, limiting assessment of sustained clinical benefit. Publication bias also remains possible because studies with positive findings may be more likely to reach publication. Additionally, the literature search was completed in September 2024, therefore, studies published after this date were not captured. The interventions represented in this review should consequently not be interpreted as representative of the full spectrum of nonsurgical CSCI care in the United States or internationally.

Implications for Clinical Practice

The findings highlight continued interest in multimodal and individualized approaches to CSCI management. However, the therapies identified in this review should be considered investigational adjuncts rather than established alternatives to standard care.

BCI-FES systems and biologic or pharmacologic therapies demonstrated preliminary signals of benefit but require additional validation, larger studies, and longer follow-up before their roles in routine clinical care can be established.

Future clinical studies should incorporate standardized functional and patient-reported outcome measures to facilitate evaluation of treatment effects and inform the development of evidence-based CSCI rehabilitation strategies.

## Conclusions

This scoping review mapped the current United States literature evaluating nonsurgical interventions for adult cervical spinal cord injury. The available evidence suggests that neuromodulation, electrical stimulation, biologic therapies, pharmacologic agents, and rehabilitation-based interventions have demonstrated encouraging short-term neurophysiological and functional findings in early clinical studies. However, the literature remains limited by small sample sizes, heterogeneous methodologies, inconsistent outcome measures, and limited long-term follow-up. Consequently, these findings should be interpreted as areas for further investigation rather than evidence of comparative effectiveness or support for treatment recommendations. Standardized multicenter studies with clinically meaningful outcome measures and longer follow-up are needed to clarify the role of these therapies within comprehensive cervical spinal cord injury rehabilitation.
